# Synchronous Bilateral In Situ Solid Papillary Carcinoma of the Breast: A Report of a Rare Case

**DOI:** 10.7759/cureus.95543

**Published:** 2025-10-27

**Authors:** Rodney E Shackelford, Xiaoming Fan, Ardenne S Martin, Cletus A Arciero, Eric X Wei

**Affiliations:** 1 Pathology, Emory University School of Medicine, Atlanta, USA; 2 Pathology and Translational Pathobiology, Louisiana State University Health Shreveport (LSUHS), Shreveport, USA; 3 Pathology and Laboratory Medicine, University of South Alabama College of Medicine, Mobile, USA; 4 Surgery, Emory University School of Medicine, Atlanta, USA; 5 Pathology and Cell Biology, University of South Florida, Tampa, USA; 6 Pathology and Laboratory Medicine, Tampa General Hospital, Ruffolo, Hooper, and Associates, Tampa, USA

**Keywords:** bilateral breast masses, breast cancer pathology, solid papillary carcinoma in situ, synchronous bilateral breast cancer, synchronous breast cancer

## Abstract

Solid papillary carcinoma is a rare breast neoplasm characterized by neuroendocrine differentiation, a circumscribed solid growth pattern, and delicate interspersed fibrovascular cores. It typically exhibits low to intermediate nuclear atypia and generally carries an excellent prognosis, even when invasion is present. Most cases are unilateral, with bilateral occurrences being exceptionally uncommon. Here, we describe a very rare case of bilateral in situ solid papillary carcinomas of the breast in a 67-year-old woman.

## Introduction

Solid papillary carcinoma (SPC) of the breast was first described in 1956 by Maluf and Koerner [1] as a distinctive histopathologic breast lesion seen in elderly women characterized by well-circumscribed expansive solid nodules containing delicate fibrovascular cores (indicating the underlying papillary architecture) and ductal cells with low- to intermediate-grade nuclear atypia and focal sarcomatous features. Perivascular tumor cell pseudorosetting is often observed surrounding the fibrovascular cores, and tumors frequently exhibit neuroendocrine differentiation. SPCs account for less than 1% of breast cancers, have a mean presentation age of 70 years, and generally have a favorable prognosis [2]. Approximately 95% of these tumors are unilateral, with around 50% arising centrally in the retroareolar or the subareolar regions, occasionally presenting with a nipple discharge [1,2]. Bilateral SPCs are very rare, with only three cases reported in the literature [3-5]. Here, we present a case of bilateral in situ SPCs in a 67-year-old woman.

## Case presentation

A 67-year-old Caucasian woman with a previous history of triple-negative stage IIA left breast invasive ductal carcinoma, status post-lumpectomy with catheter-based radiation (MammoSite) and chemotherapy 12 years earlier, was referred for the evaluation of bilateral estrogen receptor-positive mammary carcinoma identified by needle core biopsies. The differential diagnosis of the bilateral breast lesions included ductal carcinoma in situ (DCIS) in association with a papilloma, papillary DCIS, and variants of papillary carcinoma including encapsulated papillary carcinoma and SPC. Her previous radiation therapy resulted in a left breast streptococcal infection with abscess and subsequent scar formation. The patient denied any palpable breast masses, skin changes, or nipple discharge. She had no significant family history of breast or other cancers and was never on hormone therapy. Palpation of the patient's breasts revealed no obvious lumps. 

**Figure 1:**
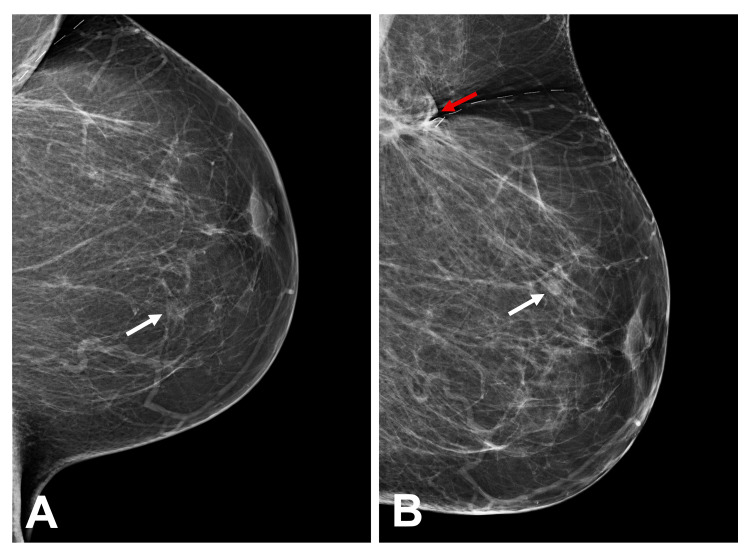
Tomosynthesis imaging of the patient's left and right breasts Tomosynthesis imaging of the patient's left (A) and right (B) breasts demonstrates bilateral scattered fibroglandular densities, a focal asymmetry in the right breast (red arrow), and bilateral oval masses with circumscribed margins. The masses are located in the middle region of the left breast at 3 o'clock and the right breast at 10 o'clock, each approximately 8 cm from the nipple (white arrows).

Tomosynthesis imaging of the patient's breasts revealed bilateral scattered fibroglandular densities with focal asymmetries in the right breast and bilateral oval masses with circumscribed margins in the middle region of the left breast at 3 o'clock and right breast at 10 o'clock (Figure 1). 

**Figure 2:**
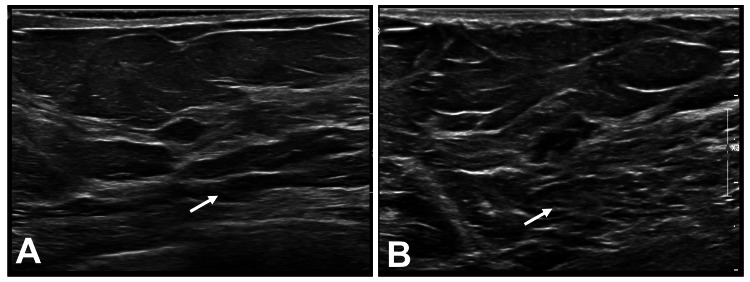
Ultrasound of the patient's left and right breasts Ultrasound of the patient's left (A) and right (B) breasts shows bilateral BI-RADS category 4 irregular, indistinct, hypoechoic masses measuring 6 mm and 30 mm in the left breast and 11 mm and 80 mm in the right breast, respectively (white arrows). BI-RADS: Breast Imaging-Reporting and Data System

To further characterize these lesions, an ultrasound was performed. Ultrasound analysis of the left breast revealed that the 3 o'clock lesion measured 6 mm and was 30 mm from the nipple, while the right lesion measured 11 mm and was 80 mm from the nipple (Figure 2). Both lesions appeared as irregular, indistinct, hypoechoic masses and were classified as category 4 in the Breast Imaging-Reporting and Data System (BI-RADS), licensed under the Creative Commons Attribution-NoDerivatives 4.0 International License (CC BY-ND 4.0) [6]. 

**Figure 3:**
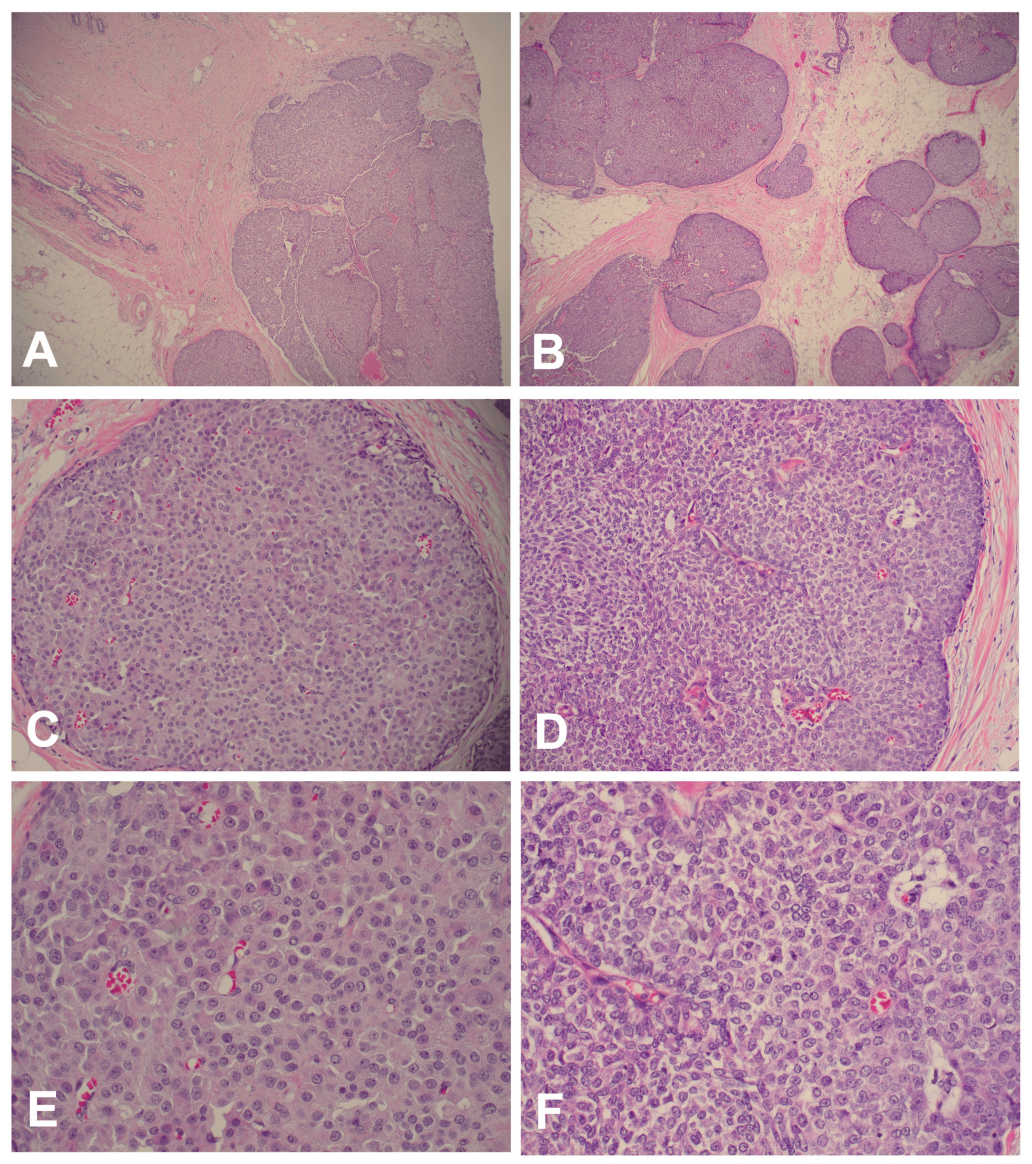
H&E staining of the bilateral SPCs Low-power H&E stains of the left (A) and right (B) breast lesions, medium-power H&E stains of the left (C) and right (D) lesions, and high-power H&E stains of the left (E) and right (F) lesions are shown. The bilaterally intact myoepithelial layers and the delicate fibrovascular cores are evident in the high-power images (E and F). H&E: hematoxylin and eosin; SPC: solid papillary carcinoma

Right and left segmental mastectomies were performed. Interestingly, histological analyses revealed that both bilateral lesions consisted of rounded nodules of moderately pleomorphic epithelioid cells with focal sarcomatous features and interspersed delicate fibrovascular cores showing focal perivascular tumor cell pseudorosetting. The nodular areas were bounded by a histologically discernible intact myoepithelial layer, suggestive of bilateral in situ SPCs (Figure 3).

**Figure 4:**
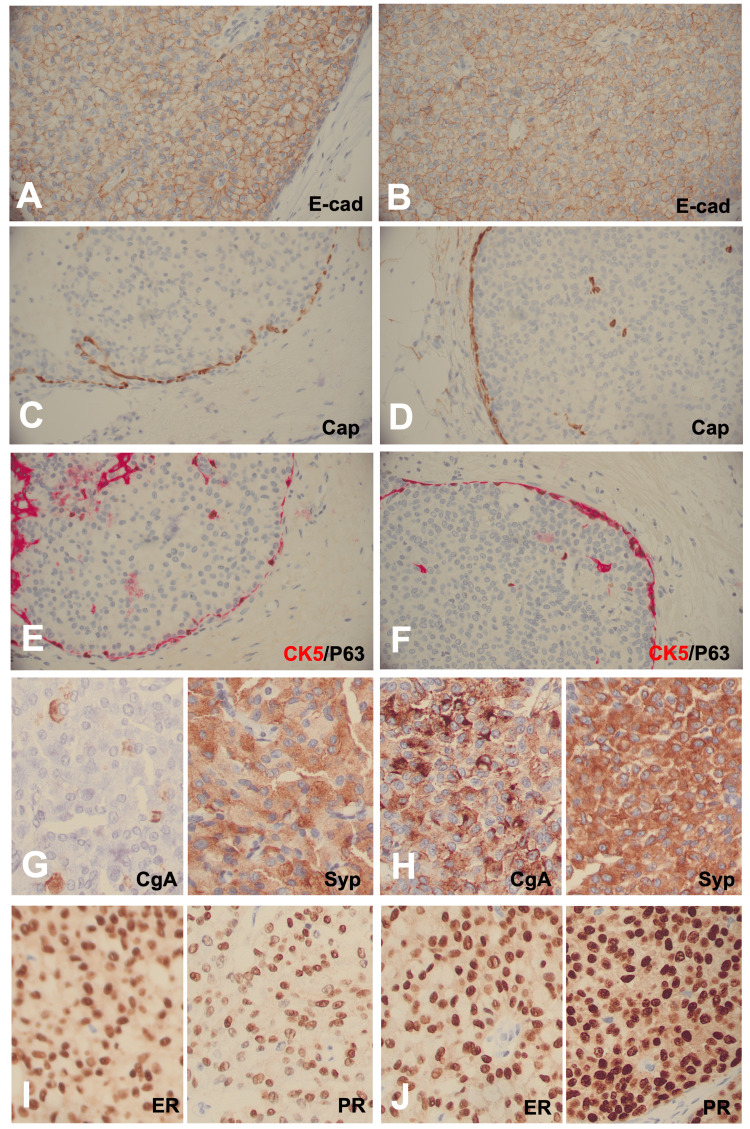
IHC analysis of the bilateral SPCs The tumor cells show retained crisp membranous staining of E-cad in the left (A) and right (B) lesions. Cap (C and D) and P63/CK5 (E and F) staining demonstrate intact myoepithelial layers in both the left and right lesions. Positive staining for CgA and Syp in the left (G) and right (H) lesions indicates neuroendocrine differentiation. Further biomarker analysis shows ER and PR positivity in the left (I) and right (J) lesions. IHC: immunohistochemical; SPC: solid papillary carcinoma; E-cad: E-cadherin; Cap: calponin; CgA: chromogranin A; Syp: synaptophysin; ER: estrogen receptor; PR: progesterone receptor

To further analyze the case, immunohistochemical stains for p63, CK5, and calponin were performed to assess the presence of an intact myoepithelial layer. E-cadherin was used to highlight the membranous staining typically seen in most breast carcinomas. Additionally, chromogranin A and synaptophysin were used to identify the tumors for neuroendocrine differentiation. The p63 and CK5 immunostains were a dual stain, with p63 staining brown and CK5 staining red. As shown in Figure 4, the p63, calponin, and CK5 immunostains reveal an intact myoepithelial layer in both the right and left breast SPCs, with the calponin and CK5 immunostains staining the myoepithelial cell plasma membranes and the brown p63 immunostain highlighting the myoepithelial cell nuclei. Additionally, the E-cadherin immunostain revealed crisp, membranous staining, consistent with that of a breast carcinoma. Lastly, the right SPC was immunopositive for both synaptophysin and chromogranin, while the left SPC was immunopositive for synaptophysin and immunonegative for chromogranin. Biomarker analyses on the right breast estrogen and progesterone receptors were 3+ 99% and 73% immunopositively, respectively, while the left breast biomarkers were 3+ 87% and 66%. Based on the histology and immunohistochemical results, a diagnosis of bilateral in situ SPCs was given. HER2/neu biomarker immunostaining was not performed, as this testing is not required for in situ breast cancer. 

## Discussion

First identified in 1956, the World Health Organization (WHO) Classification of Tumours of the Breast defines SPC as a distinctive form of papillary carcinoma characterized by closely apposed expansive, cellular nodules [1,2,7]. SPCs constitute less than 1% of breast lesions, usually present in older women (seventh decade), and about 50% show neuroendocrine differentiation, with one study demonstrating that SPCs account for about 43.2% of all breast tumors with neuroendocrine differentiation [1,2]. Approximately 95% of SPCs are unilateral. Bilateral SPCs are exceptionally rare, with only three cases reported (Table 1).

**Table 1:** Summary of the previously reported cases of bilateral SPCs of the breasts SPC: solid papillary carcinoma; IHC: immunohistochemical

Studies	Age	SPC tumor location and size	Tumor data	Metastases	Outcome
Yoshimura et al. [3]	73	Right: 17 mm	SPC with invasive ductal carcinoma	Right axilla	No recurrence in 5 years
		Left: 8 mm (2 lesions)	SPC with invasive mucinous carcinoma (both)		
Moon et al. [4]	69	Right: 8 mm	SPC with invasion measuring 9×8×5 mm	Right axilla	No recurrence at 9 months
		Left: 12 and 9 mm (2 lesions)	SPC with invasion measuring 66×35×25 mm		
Kılınç et al. [5]	74	Right: 18 mm	Invasion identified by SPC geographical borders and no myoepithelial layer by IHC analysis	No	No recurrence in 2 years
		Left: 20 mm			

Typically, SPCs show multiple circumscribed nodules containing epithelioid and occasionally sarcomatous cells with low-grade nuclei, occasional focal extracellular mucin, and interspersed thin delicate fibrovascular cores. Cellular palisading (pseudorosettes) is often seen around the fibrovascular cores, accompanied by hyalinization. Likely SPCs originate from expanded ducts and usually involve the central region of the breast. SPCs can be misdiagnosed as lesions such as florid ductal hyperplasia, lobular neoplasia, encapsulated papillary carcinoma, and low nuclear grade DCIS. SPCs have a favorable prognosis, especially in cases with only in situ histology, and exhibit exceedingly uncommon metastases. It is therefore important to distinguish them from other mammary carcinomas [1,2]. 

SPC is considered an in situ lesion when the tumor nodules have smooth boundaries combined with a distribution pattern reflecting the involvement of the underlying glandular tree. Thus, the myoepithelial cells may be fully retained, attenuated, or absent in an in situ SPC. Similarly, the myoepithelial cell layer can be attenuated or absent in the SPC internal fibrovascular cores. Interestingly, SPC basement membrane loss correlates with increased carbonic anhydrase IX expression [8]. The WHO recommends that if uncertainty exists regarding the presence of invasion in a specific case, the tumor should be staged as in situ disease and all diagnoses should designate whether the lesion is in situ or invasive [1,2,7]. Interestingly, the case presented here was bilaterally in situ with easily identified, non-attenuated myoepithelial cell layers (Figures 3-4). 

Invasive SPC is diagnosed by identifying SPC with extralobular stroma showing irregular, geographic/jigsaw-like tumor contours accompanied by stromal desmoplasia, vascular invasion, normal gland engulfment, and/or fat infiltration, which usually does not follow the underlying glandular tree pattern. Additionally, SPCs can often show heterologous invasive components consisting of invasive mucinous, ductal, SPC, and lobular carcinomas and combinations of all these carcinoma types. The staging of invasive SPC is determined by the size of the invasive component, with Nottingham grading and estrogen and progesterone receptor and HER2/neu studies performed on the invasive tumor component [1,2,7]. Immunohistochemistry can be useful in the diagnosis and analysis of SPCs. Approximately 50% of SPCs are immunopositive for neuroendocrine markers, such as chromogranin and synaptophysin, reactivities that are rare in DCIS and florid ductal hyperplasia. SPCs have interspersed thin fibrovascular cores and some with spindle cell morphology, which are lacking in solid-type DCIS. SPCs are also immunonegative for high-molecular-weight cytokeratins; thus, CK5/6 immunonegativity will distinguish SPCs from florid ductal hyperplasia. SPCs show solid growth patterns, while encapsulated papillary carcinoma reveals a complex expansible partially cystic mass with filiform branching papillae lined by columnar cells without neuroendocrine differentiation. SPCs also commonly show a luminal phenotype and are typically estrogen and progesterone receptor immunopositive and HER2/neu negative. Tall cell carcinoma with reverse polarity shows apical nuclei and a triple-negative phenotype with high-molecular-weight cytokeratin and calretinin expression. Neuroendocrine tumor or carcinoma of the breast demonstrates morphological features similar to those originating in the lung or gastrointestinal (GI) tract, very distinct from SPCs which may have some neuroendocrine differentiation. Lastly, myoepithelial markers, such as p63, calponin, and smooth muscle actin, can help identify in situ vs. invasive lesions [1,2,6]. At the molecular level, SPCs show a transcriptomic gene expression pattern closely resembling mucinous B carcinomas and loss of chromosome 16q, gain of chromosomes 1p and 16p, and higher expression of genes associated with neuroendocrine differentiation, including RET, ASCL1, and DOC7 [1,2].

## Conclusions

We present a rare case of bilateral in situ SPC, characterized by easily identifiable myoepithelial cell layers and absence of invasion. However, a reliable conclusion regarding the biological behavior of these rare lesions cannot be drawn. It remains possible that bilateral SPC may carry an increased risk for invasive disease, and further investigation of additional cases is required to establish definitive conclusions.
